# Neuropeptides and Their Roles in the Cerebellum

**DOI:** 10.3390/ijms25042332

**Published:** 2024-02-16

**Authors:** Zi-Hao Li, Bin Li, Xiao-Yang Zhang, Jing-Ning Zhu

**Affiliations:** 1 State Key Laboratory of Pharmaceutical Biotechnology and Department of Physiology, School of Life Sciences, Nanjing University, Nanjing 210023, China; 602022300020@smail.nju.edu.cn (Z.-H.L.); jnzhu@nju.edu.cn (J.-N.Z.); 2 Women and Children’s Medical Research Center, The First Affiliated Hospital of Nanjing Medical University, Nanjing 210029, China; 3 Institute for Brain Sciences, Nanjing University, Nanjing 210023, China

**Keywords:** neuropeptide, cerebellum, cerebellar development, cerebellar ataxia, autism, motor coordination, motor learning, synaptic plasticity

## Abstract

Although more than 30 different types of neuropeptides have been identified in various cell types and circuits of the cerebellum, their unique functions in the cerebellum remain poorly understood. Given the nature of their diffuse distribution, peptidergic systems are generally assumed to exert a modulatory effect on the cerebellum via adaptively tuning neuronal excitability, synaptic transmission, and synaptic plasticity within cerebellar circuits. Moreover, cerebellar neuropeptides have also been revealed to be involved in the neurogenetic and developmental regulation of the developing cerebellum, including survival, migration, differentiation, and maturation of the Purkinje cells and granule cells in the cerebellar cortex. On the other hand, cerebellar neuropeptides hold a critical position in the pathophysiology and pathogenesis of many cerebellar-related motor and psychiatric disorders, such as cerebellar ataxias and autism. Over the past two decades, a growing body of evidence has indicated neuropeptides as potential therapeutic targets to ameliorate these diseases effectively. Therefore, this review focuses on eight cerebellar neuropeptides that have attracted more attention in recent years and have significant potential for clinical application associated with neurodegenerative and/or neuropsychiatric disorders, including brain-derived neurotrophic factor, corticotropin-releasing factor, angiotensin II, neuropeptide Y, orexin, thyrotropin-releasing hormone, oxytocin, and secretin, which may provide novel insights and a framework for our understanding of cerebellar-related disorders and have implications for novel treatments targeting neuropeptide systems.

## 1. Introduction

The cerebellum has been well established as a subcortical center for motor coordination and motor learning, while its nonmotor functions, e.g., reward and cognition, have attracted increasing attention [1]. The multifaceted role of the cerebellum relies on its well-organized structure and circuitry during development [2]. Neurons in the cerebellum are derived from two germinal zones: the cerebellar ventricular zone (VZ) and the cerebellar rhombic lip (CRL) [3]. The progenitors in the cerebellar VZ give rise to GABAergic inhibitory neurons, including Purkinje cells (PCs), Golgi cells, basket cells, stellate cells, candelabrum cells, and small neurons in the cerebellar nuclei (CN) in mammals [2]. Likewise, the neuroepithelial cells from the CRL produce glutamatergic excitatory neurons, including granule cells (GrCs), unipolar brush cells, and large projection neurons in the CN [2]. These GABAergic and glutamatergic neurons are arranged into a three-layer folded cortex (the molecular, PC, and GrC layer, from superficial to deep) with paired sets of CN (fastigial, interposed, and dentate nucleus, from medial to lateral) at its base. During the neuronal morphogenesis and positioning in the cerebellum, PC maturation and GrC proliferation and migration have been recognized as critical events [4]. PCs migrate from the VZ toward the outer surface of the cerebellum, form a single lamina, i.e., the PC layer, and develop large and complex dendritic trees at the early postnatal stage [5]. In parallel, CRL gives rise to cerebellar GrC progenitors, which proliferate and migrate to form the external granular layer (EGL), completely covering the embryonic cerebellar surface. GrC progenitors proliferate in the outer EGL to generate the postmitotic GrCs that invade the inner EGL and migrate radially through the molecular layer, past the PC layer, to eventually settle in the internal granular layer (IGL) [6]. By the end of the third postnatal week, EGL cells have completed migration to the IGL, the GrC layer in the mature cerebellum [7].

The maturation of the cerebellum leads to the final setting of the cerebellar circuitry. The cerebellar neurons receive two major types of afferent inputs—mossy fibers (MFs) and climbing fibers (CFs)—both of which use glutamate as their primary neurotransmitters [8]. The MFs, originating from many sources in the brainstem and spinal cord, synapse with GrC dendrites in the GrC layer [8]. The GrCs then send their axons, i.e., the parallel fibers (PFs), up to the molecular layer, and relay MF inputs to the distal dendrites of PCs [8]. Each PC receives functionally weak but numerous (e.g., 100,000 in mice) PF synapses [8]. In contrast, the CFs arise exclusively from neurons in the inferior olive (IO), and innervate the proximal dendrites of PCs [8]. In the mature cerebellum, each PC is traditionally assumed to be mono-innervated by a single strong CF because of the axonal competition during development [9]. In addition, multibranched PCs have recently been found to receive more than one CF afferent input [10]. In either case, PCs integrate information from excitatory inputs of PFs and CFs and local inhibitory input from molecular layer interneurons, e.g., basket cells and stellate cells, and generate the sole output of the cerebellar cortex [8]. Finally, CN neurons receive inhibitory inputs from PCs as well as direct inputs through MF and CF collaterals, constituting the cerebellar final outputs [8]. Through this input–output organization, the cerebellum serves as a unique hub in the central nervous system to not only fine-tune motor activity but also modulate nonmotor processes. Moreover, multiple forms of plasticity have been revealed at various synaptic and extrasynaptic sites in the cerebellar cortex and nuclei, which have been considered to be neural substrates for motor learning [11].

**Figure 1:**
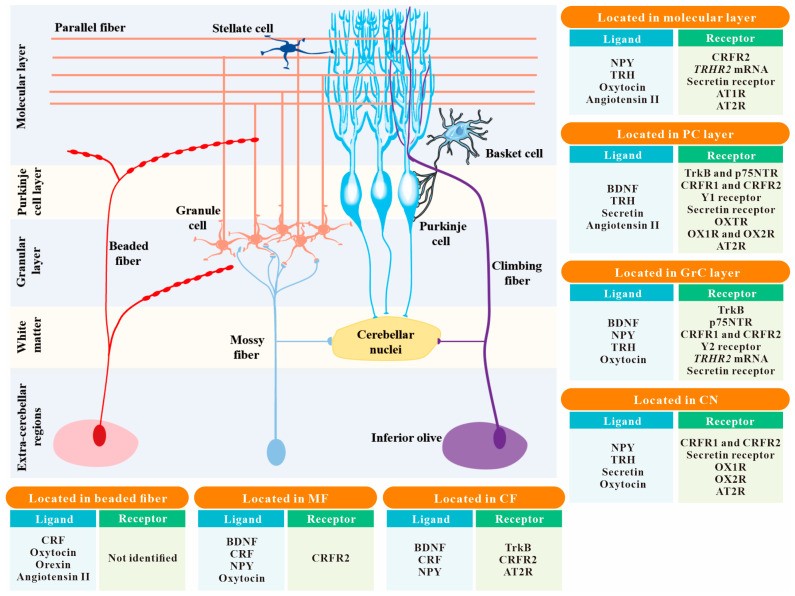
The spatial distribution of 8 common neuropeptides and their receptors within the cerebellar circuitry. The localizations of neuropeptides and their receptors in the 7 main structures of the cerebellum, including the molecular layer, PC layer, GrC layer, CN, MF, CF, and beaded fiber, are summarized in the insert boxes. Neuropeptides not mentioned in this review are not listed in these boxes. Abbreviation: PC, Purkinje cell; GrC, granule cell; CN, cerebellar nuclei; MF, mossy fiber; CF, climbing fiber; BDNF, brain-derived neurotrophic factor; CRF, corticotropin-releasing factor; NPY, neuropeptide Y; TRH, thyrotropin-releasing hormone; TrkB, tropomyosin-related kinase B (one of the BDNF receptors); p75NTR, p75 neurotrophin receptor (a low-affinity receptor for BDNF); CRFR1 and CRFR2, CRF receptor 1 and CRF receptor 2; Y1 receptor and Y2 receptor, neuropeptide Y receptor 1 and neuropeptide Y receptor 2; TRHR1 and TRHR2, TRH receptor 1 and TRH receptor 2; OXTR, oxytocin receptor; OX1R and OX2R, orexin type 1 receptor and orexin type 2 receptor; AT1R and AT2R, angiotensin II type 1 receptor and angiotensin II type 2 receptor.

In addition to the classic small-molecule neurotransmitters, e.g., glutamate and GABA, neuropeptides have also been reported to act as critical mediators of intra- and extra-cerebellar circuit connectivity. Ito has summarized 22 different types of neuropeptides distributed in the cerebellum [12]. A more recent study has determined the expression of neuropeptides in the cerebellum from birth to adulthood by using a semiquantitative peptidomic approach, and identified a total of 33 neuropeptides [13]. In contrast to classic neurotransmitters, the synthesis, release, and removal of neuropeptides exhibit unique features. Firstly, neuropeptides are synthesized in the somata of neurons and transported down the axon, whereas small-molecule neurotransmitters are synthesized and stored in the terminal for fast release [14]. Secondly, a longer train of action potentials and more calcium influx are required for neuropeptides to be released into the synaptic cleft, while a single action potential may give rise to the release of amino acids [14]. In addition, neuropeptides are generally degraded by peptidases located on cytomembranes of neurons and glia. Notably, the concentration of these peptidases is relatively low, so neuropeptides may diffuse from their release site to remote receptors at a long range, allowing them to affect larger populations of neurons [14]. Unlike neuropeptides, the synaptic effects of small-molecule neurotransmitters are terminated by quick transport back into nerve terminals [14]. In the cerebellum, multiple neuropeptides have been reported to be essential for cerebellar development and modulation of neuronal activity and synaptic plasticity. Moreover, neuropeptides have also been implicated in the pathological changes in the cerebellum and may serve as potential therapeutic agents in cerebellum-related disorders. In this review, we focus on 8 of the top 15 neuropeptides with great research progress over the last two decades (2004–2024; Table 1) that are most relevant to neurodegenerative and/or neuropsychiatric disorders, including brain-derived neurotrophic factor (BDNF), corticotropin-releasing factor (CRF), angiotensin II, neuropeptide Y (NPY), orexin, thyrotropin-releasing hormone (TRH), oxytocin, and secretin, and summarize their anatomical distributions and functions in the cerebellum (Figure 1).

## 2. BDNF

BDNF, discovered in 1982, is a member of the neurotrophic family of growth factors [15]. Synthesis and maturation of BDNF is a multistage process that culminates in the formation of a mature 119-amino acid protein neuropeptide [16]. BDNF is distributed in various brain regions, but the cerebellum and hippocampus have the highest expression levels [17]. In the cerebellum, *Bdnf* mRNA is restricted to the IGL and PCs, and its protein is widely distributed within the cerebellar cortex [18] (Figure 1). Moreover, cerebellar BDNF comes from extracerebellar sources via CFs and MFs [18,19,20]. Two types of receptors binding to BDNF, i.e., tropomyosin-related kinase B (TrkB) and the low-affinity p75 neurotrophin receptor (p75NTR), have been reported to be expressed abundantly in GrCs with a weaker expression in PCs of the cerebellum [19,20]. TrkB receptor is expressed in both pre- and post-migratory GrCs [19], whereas p75NTR receptor is solely expressed in pre-migratory GrC precursors in the EGL and downregulated before GrC precursors migration and differentiation [21].

BDNF is essential for cerebellar development. Firstly, many lines of evidence have indicated that BDNF promotes the survival and maturation of GrCs. In cerebellar GrC cultures, GrCs have more survival and fiber outgrowth in the presence of BDNF [22]. Mice with gene deletion of BDNF exhibit increased apoptosis of GrCs and a sparser and thinner IGL on postnatal day 8 (P8) [23]. In addition, deletion of BDNF delays the disappearance of EGL [23], indicating an impaired migration and maturation of GrCs. A recent study has also shown a critical role of p75NTR in the EGL of the cerebellum. In the cultured EGL cells from p75NTR knockout rats, the cell cycle of GrC progenitors is accelerated and thus leads to excessive progenitor proliferation within the EGL [21]. In contrast to p75NTR, TrkB receptor is abundantly distributed in mature GrCs [24], indicating that TrkB signaling may promote the maturation and maintenance of differentiated GrCs. In cultured cerebellar EGL cells whose differentiation has been initiated by signals within the cellular reaggregates of EGL, BDNF promotes neurite extension and survival of differentiated GrCs, which can be blocked by the Trk inhibitor K-252a [24]. However, gene depletion of TrkB receptor in cerebellar precursors does not reduce the number of GrCs or their excitatory synapses when EGL progenitor cells have completed their migration to the IGL by the end of the third postnatal week [25], indicating that there may be a compensatory mechanism in vivo to lead to a normal final density of GrCs in the adult cerebellum in the absence of TrkB receptor. In line with this speculation, it has been found that TrkB and the neurotrophin 3 receptor TrkC cooperate in promoting the survival of GrCs. Massive cell apoptosis of GrCs has been observed on P14 in TrkB and TrkC double-mutant mice, but not in mutant mice deficient of a single Trk receptor [26]. Secondly, BDNF signaling is involved in CF synapse elimination in the cerebellum. In BDNF knockout mice, innervation of PCs by multiple CFs fails to be fined into mono-innervation [27]. By PC-specific deletion of BDNF combined with the knockdown of BDNF receptor in CFs, it has been observed that BDNF acts retrogradely on TrkB in CFs, and facilitates the elimination of CF synapses from PC soma after P16 [27]. Finally, cerebellar BDNF signaling is essential for the development of inhibitory synapses in the cerebellum. In PC-specific BDNF knockout mice, the density of parvalbumin-positive GABAergic interneurons in the molecular layer and the density of GABAergic terminals on the PC soma are markedly reduced on P15 and P16 [27]. By cerebellum-specific deletion of TrkB, the density of GABAergic synapses decreases in both the GrC layer and molecular layer during P50–P80 [25].

Moreover, accumulating studies have highlighted a direct and critical role of BDNF in regulating cerebellar neuronal excitability, synaptic transmission, and synaptic plasticity. In the cerebellum, BDNF has been found to elicit rapid depolarizing responses in GrCs and PCs by activating Na^+^ channels coupled to TrkB receptor [28]. Several independent studies have shown that BDNF exerts dual actions to modulate GABAergic transmission. By activating potassium–chloride cotransporter 2 (KCC2) coupled to TrkB signaling, BDNF reduces the efficacy of GABAergic transmission in PCs in rat cerebellar slices [29]. Another study has shown that GABAergic transmission from PCs is potentiated by co-activating TrkB and Src (a member of nonreceptor tyrosine kinases) [30]. In addition, BDNF may also modulate GABAergic transmission by regulating the amount of GABA_A_ receptors, as well as the synthesis and transport of GABA. The hyperammonemia-induced overexpression of GABA_A_ receptors, GABA-synthesizing enzymes, and GABA transporters within the cerebellum can be reversed by the blockage of the BDNF-TrkB pathway [31]. Notably, BDNF also plays a fundamental role in the modulation of cerebellar synaptic plasticity. In cerebellar PCs, BDNF can induce ionic plasticity, a form of synaptic plasticity unique to inhibitory neurotransmission, which is manifested as a decrease in synaptic strength of GABAergic transmission due to a reduced transmembrane CI^−^ gradient mediated by the coupling between GABA_A_ receptor and KCC2 [32]. Furthermore, BDNF has been reported to be required for vesicle docking and to contribute to short-term plasticity, such as paired-pulse facilitation, at the PF–PC synapses of the cerebellum [33].

The suppression of BDNF expression is a potentially momentous phenomenon in many neurodegenerative disorders caused by abnormal expansion of tri-nucleotide (CAG) repeats encoding polyglutamine (polyQ), such as spinocerebellar ataxias (SCAs). The cerebellum of individuals with SCA type 6 (SCA6) shows reduced *Bdnf* mRNA expression and abnormal localization of BDNF protein [34]. Moreover, mice with a targeted gene deletion of BDNF show decreased TrkB signaling in GrCs and PCs and exhibit a wide-based ataxic gait [23], indicating that the impairment of BDNF-TrkB signaling pathway may not only result from polyQ expansion but also contribute to the onset of ataxia symptoms. Several lines of evidence have suggested that an enhancement of the cerebellar BDNF pathway may provide therapeutic strategies for the treatment of spinocerebellar ataxias. It has been reported that chronic treatment with aminopyridines, which are associated with increased levels of cerebellar BDNF, can correct early dysfunction and delay neurodegeneration in SCA1 mice [35]. Moreover, an increase in the production of BDNF may act as a primary mechanism by which mesenchymal stem cell therapy promotes PC survival and improves ataxic motor dysfunction in Lurcher mutant mice [36]. In addition to cerebellar ataxias, BDNF plays crucial neuroprotective and immunomodulatory roles in other pathological situations. In rat cerebellar GrC cultures, BDNF administration markedly reduces the glutamate-induced cell death of GrCs [22]. Rat pups with subcutaneous administration of estradiol on P4 increase cerebellar *Bdnf* mRNA and protein levels, and attenuate ethanol-induced PC loss and motoric impairment, indicating the involvement of BDNF pathway in neuroprotective effects of estrogen against ethanol toxicity in the developing cerebellum [37]. Enhanced cerebellar BDNF expression has also been reported to contribute to improving high-fat diet-induced dysregulations in cytokines in the cerebellum with exercise training and melatonin administration [38]. In addition, BDNF underlies the beneficial effects of environmental enrichment [39], which has been known to be highly related to the reduction in proinflammatory cytokines and chemokines in the brain [40]. Therefore, the cerebellar BDNF signaling pathway may provide potential therapeutic targets for cerebellar-related neuroimmune and neurodegenerative disorders.

## 3. CRF

CRF, chemically identified in 1981 and also known as corticotropin-releasing hormone (CRH), is a 41-amino acid polypeptide that is synthesized and released by neurons in the paraventricular nucleus of the hypothalamus, as well as those in broad extrahypothalamic brain regions, including IO, the sole origin of cerebellar CFs [41,42] (Figure 1). CRF is well known to be implicated in autonomic and behavioral components of the stress response [42]. It has been reported that *Crf* mRNA in the IO is upregulated shortly in stress-induced motor responses, and IO-specific knockdown of CRF is sufficient to induce motor deficiency under either normal [43] or challenging conditions [44]. In addition to CFs, CRF may also be expressed in MFs and present as a beaded plexus lying parallel to the pial surface, above and subadjacent to the PC layer [45]. Among these populations of CRF-immunopositive afferent inputs, IO-derived CFs are the biggest source. CRF immunoreactive somata are distributed throughout all divisions of the IO [45]. Postmortem human cerebellum obtained from patients with olivopontocerebellar atrophy showed a significant decrease in cerebellar CRF concentration [46], indicating a role of CRF signaling in cerebellar ataxias. In fact, the CF-derived CRF promotes cerebellar motor coordination. By using retrograde tracing, immunohistochemistry, and whole-cell patch clamp recordings, the direct CRFergic projections from the IO have been identified to excite glutamatergic projection neurons in the cerebellar interposed nucleus via two CRF receptors, CRFR1 and CRFR2, coupled to inward rectifier K^+^ channels and hyperpolarization-activated cyclic nucleotide-gated channels [43]. In addition, microinjection of CRF into the bilateral interposed nuclei not only promotes motor performances in normal rats but also ameliorates ataxia-like motor abnormalities induced by the IO-specific *Crf* mRNA downregulation and 3-acetylpyridine (3-AP) administration [43]. These results strongly suggest that CRF in the olivocerebellar system may hold a key position in the pathophysiology and treatment of cerebellar ataxia.

In addition to the excitatory effects on neurons in the CN, CRF signaling is essential for neuronal activity and plasticity modulation of PCs in the cerebellar cortex. CRF increases PC excitability by modulating sodium, potassium, and hyperpolarization-activated action currents [47]. Antagonizing CRF receptors by α-helical CRF or astressin blocks long-term depression (LTD) of PF–PC [48] and CF–PC [49] synaptic transmissions. In contrast to improving LTD, which favors the suppression of glutamatergic transmission in PCs, CRF has also been reported to promote CF–PC glutamatergic transmission, manifested by an enhancement in complex spike activity of PCs, via the presynaptic autoreceptor CRFR2 and the underlying PKA signaling in CF terminals [50]. These results indicate that CRF may regulate the strength of excitatory inputs to PCs bidirectionally. Moreover, CRF can block facial stimulation-induced LTD at inhibitory synapses between the molecular layer interneurons and PCs by triggering long-term potentiation (LTP) at these synapses via CRFR1 and its underlying PKC signaling pathway [51]. In addition to the modulatory effects on synaptic inputs to PCs, GrC-specific knockout of CRFR1 has also been shown to affect MF–GrC transmissions, manifested by converting the high-frequency MF stimulation-induced LTP into LTD [52]. Consistent with its significant roles in plasticity modulation, cerebellar CRF signaling is actively involved in motor learning and adaptation. Mice with GrC-specific CRFR1 deletion show accelerated eyeblink conditioning learning [52]. Intriguingly, in line with the result that stress disrupts eyeblink conditioning in humans [53,54], depletion of CRFR1 in rodents rescues animals from the hazardous effects of early life stress or prolonged stress on learning [55,56]. Furthermore, the transcription and expression of CRF within the IO are influenced by optokinetically evoked IO discharge and may contribute to optokinetic adaptation [57].

CRF and its receptors are present during the critical period of cerebellar development and sustainably expressed throughout the postnatal period [58,59]. The cellular localization of CRFR1 immunoactivity in PCs translocates from the apical processes at birth (P0), to the somata (P3), and finally to the primary dendrites (P9) during PC development in the postnatal mouse cerebellum [60]. In contrast to CRFR1, CRFR2 immunoreactivity is predominantly localized to the somata and axon hillocks of cerebellar PCs, and is not expressed in large quantities until P12 [61]. A truncated isoform of CRFR2 is primarily expressed in presynaptic localization within the cerebellar cortex [62]. It has been proposed to be involved in the development of extracerebellar afferents [63]. Several lines of in vitro evidence indicate the role of CRF signaling in the development of PCs. Applying CRF to rat cerebellar slice cultures increases the density of dendritic spines on PCs, which can be blocked entirely by combined administration of selective antagonists for CRFR1 and CRFR2 [64]. Moreover, more dendritic outgrowth and elongation are observed in PCs exposed to CRF intermittently [65]. Instead, exposure to CRF constantly inhibits the dendritic outgrowth of PCs [65].

## 4. Angiotensin II

Angiotensin II is a bioactive octapeptide of the renin–angiotensin system (RAS) involved in controlling blood pressure and maintaining fluid homeostasis that was first isolated in 1954 [66]. This peptide can also be produced within the central nervous system and serve as a neurotransmitter and neurohormone [67]. In an early study, Changaris et al. [68] found that fibers with angiotensin II immunoprecipitation traverse through the white matter and diverge within the GrC layer, terminating on the somata of PCs in the rat cerebellum (Figure 1). Detecting angiotensin II distribution in the rat cerebellar cortex by immunogold staining [69] has revealed that angiotensin II immunoreactivity is prominent in cerebellar PCs, GrCs, basket cells, and stellate cells. At the subcellular level, the peptide is clearly localized in the transcriptionally active euchromatin of nuclei, suggesting a role of angiotensin II in regulating gene transcription [69]. By comparing their global transcriptional data with additional published datasets, Elkahloun and Saavedra have reported that the selective blocker for angiotensin II type 1 receptor (AT1R), candesartan, can protect from the alterations in aging- and senescence-related gene expression in the cerebellum of old mice [70]. As expected, angiotensin II receptors were also found in the cerebellum using an in vivo cryotechnique, albeit with distinct expression patterns. Immunoreactivity of AT1R is highest in the outer molecular layer and largely overlaps with Bergmann glia in the mouse cerebellum [71] (Figure 1). Unlike AT1R, AT2R immunoreactivity in the rat cerebellum is primarily associated with the PC layer and the CNs, rather than the molecular layer [72]. In addition, the levels of two angiotensin II receptors are both time-dependently modulated under hypoxia in the cerebellum [71], which might be related to the role of angiotensin II and its receptors in regulating cerebellar hemoperfusion.

During the critical period in the postnatal development of the cerebellum, *At2r* mRNA is initially discretely localized in neurons of the IO and translated into AT2R in situ, and then transported through CFs to the PC layer of the cerebellar cortex [73]. The result suggests a role of AT2R in the development of the IO–cerebellar pathway [73]. In microexplant cultures of the cerebellum from three-day-old rats, specific activation of the AT2R induces major morphological changes, including neurite outgrowth and cell migration, two important processes in the organization of the various layers of the developing cerebellum [74].

Angiotensin II has also been reported to be involved in regulating neuronal excitability and synaptic transmission within the cerebellum. Microiontophoretic application of angiotensin II specifically suppresses the spontaneous firing of PCs and enhances the inhibition of GABA, which can be antagonized by a specific GABA antagonist, bicuculline methochloride [75]. In addition, previous studies have suggested that angiotensin II and its receptors are emerging as critical players in neuroinflammation in the cerebellum. In cerebellar astroglial cultures, angiotensin II administration induces a robust inhibitory effect on *Il-10* mRNA expression [76]. Moreover, angiotensin II-AT1R signaling induces cyclooxygenase 2 expression to stimulate proinflammatory and mitogenic actions in cerebellar astrocytes in vivo, which potentially contributes to central sympathetic overactivity and elevates blood pressure [77]. In contrast to AT1R, several findings suggest a role for AT2R in development and cognitive function [78]. Genetic variants in AT2R abundant in the cerebellum have been identified in human X-linked mental retardation [78], with clinical symptoms associated with autistic spectrum disorder (ASD) [79]. Notably, central angiotensin II is involved in the positive regulation of systemic oxytocin [80], which could influence the connections between the cerebellum and forebrain structures associated with ASD [81].

## 5. NPY

Discovered in 1982, NPY is a 36-amino acid neuropeptide that belongs to the family of pancreatic polypeptides, along with pancreatic polypeptide and peptide YY [82]. NPY has a widespread distribution throughout the central nervous system and is most prevalent in the cortical and limbic regions, as well as the cerebellum [83] (Figure 1). Several independent studies have shown a species difference in the distribution of NPY within the cerebellum. NPY immunoactivity is exclusively expressed in the PC layer in ducks [84], while in humans, NPY immunoreactivity is observed in GrCs and neurons within the cerebellar dentate nucleus [85]. Moreover, NPY immunoactivity is also present in CFs and MFs in rats [86]. Two NPY functional receptors, Y1 and Y2, are expressed in the cerebellum. In situ hybridization and immunohistochemistry staining in rats show that Y1 receptor is exclusively expressed in PCs, while Y2 receptor is mainly expressed in GrCs of the IGL [87]. Notably, the expression of NPY and its receptors in the cerebellum fluctuates with cerebellar development. In rats, the levels of NPY immunoreactivity in the IO are transiently upregulated around the end of the first postnatal week and decrease progressively to undetectable levels in the adult [88]. Consistently, the expression of cerebellar NPY receptors is at very low levels in the embryo and elevated to a peak on P10 [87], further indicating a potential role of NPY in cerebellar postnatal development.

Several studies have shown a potential therapeutic effect of NPY on SCA3, also known as Machado–Joseph disease, characterized by motor coordination impairment and cerebellar neurodegeneration. Recently, Duarte-Neves et al. reported that NPY levels decreased in the cerebellum of two SCA3 patients and a mouse model of SCA3 [89]. Furthermore, they also observed that overexpression of NPY could preserve the cerebellar volume and GrC layer thickness, reduce the number of mutant ataxin 3 aggregates, and ameliorate motor coordination impairment [89]. The underlying mechanism may be related to the NPY-mediated increase in BDNF levels and decrease in inflammatory markers [89]. The neuroprotective and therapeutic effects of viral vector-mediated NPY overexpression in SCA3 mice can be reproduced by noninvasive intranasal infusion of NPY [90], suggesting a translational potential of NPY as a pharmacological intervention in SCA3 patients.

## 6. Orexin

Orexin, also known as hypocretin, is a neuropeptide discovered in 1998 that is produced by a small group of neurons exclusively localized to the lateral hypothalamic area (LHA) and perifornical area (PFA), with extensive projections within the central nervous system, including the cerebellum [91,92,93] (Figure 1). It is widely known that the loss of orexin-producing neurons causes narcolepsy in humans and rodents [93,94]. Therefore, orexin function has long been implicated in sleep–wake regulation [93]. However, narcolepsy is typically accompanied by cataplexy, which is a sudden loss of muscle tone in response to strong emotion [95], indicating that orexin may also exert direct control of somatic motor functions. Although two types of orexin receptors, orexin 1 receptor (OX1R) and orexin 2 receptor (OX2R), have been reported to be expressed in the CN in the early 2000s [96,97], the impact of orexin on the cerebellum and motor control was largely unknown until a decade later. Zhu and Wang’s group reported for the first time that orexin excites CN [98] and major subnuclei (such as lateral and inferior) in the vestibular nuclear complex [99], which are also well known as transitional nuclei of the vestibulocerebellum. A dual ionic mechanism involving both Na^+^-Ca^2+^ exchangers and inward rectifier K^+^ channels underlies the excitatory effect of orexin on lateral vestibular nuclear (LVN) neurons [99]. Intriguingly, blockage of orexinergic inputs in LVN strongly attenuates rat motor performance during a motor challenge rather than general movements [99]. Moreover, the stronger the motor challenge faced by animals, the greater the degree of involvement of the central orexinergic system in motor control [99]. It is therefore suggested that when encountering an unexpected challenge that requires a strong motor response, orexin deficiency results in cataplexy; in other words, narcolepsy–cataplexy caused by orexin deficiency may be, in part, a consequence of loss of orexinergic modulation on the central motor system.

Accumulating evidence has highlighted a role of cerebellar orexin signaling in motor–emotional cross talk and somatic–nonsomatic integration. In rabbit cerebellar flocculus, orexin increases the rate of simple spike discharges of PCs via OX1R and may mediate the excitation of floccular PC evoked by LHA/PFA activation, which in turn adaptively controls arterial blood flow redistribution that occurs in defense behaviors via the downstream parabrachial nucleus [100]. Endogenous orexin also increases the peak amplitude of cerebellar theta oscillations related to motor learning and modulate the timing rather than the acquisition of trace eyeblink conditioning via OX1R [101]. A recent study dissects a cross-species conserved three-neuron loop, the hypothalamus–cerebellum–amygdala loop, which bridges the subcortical motor system and limbic system [102]. Rotarod running at a constant speed activates glutamatergic neurons in the cerebellar dentate nucleus that project to PKCδ^+^ neurons in the centrolateral amygdala, from which anxiolytic processes can be coordinated with movements [102]. More challenging forms of exercise, e.g., accelerating rotarod running, recruit an additional pathway, originating from orexinergic neurons in the PFA that provide an extra excitation on the dentate neurons [102]. Thus, the three-neuron loop may mediate the ameliorating effects of motor activity on anxiety at two levels of intensity, which sheds light on developing challenging exercise strategies and cerebellar orexinergic-targeted interventions for anxiety.

## 7. TRH

TRH is a tripeptide, pGlu-His-Pro-NH2, first identified in 1969 as a releasing hormone that stimulates the secretion of pituitary thyroid-stimulating hormone (TSH, also known as thyrotropin) [103]. The distribution of TRH has been studied in the rat brain using radioimmunological techniques. TRH is distributed in almost all brain regions, among which the cerebellum contains 2.1% of the total brain content of TRH [104]. TRH is distributed inhomogeneously within the cerebellum (Figure 1). In rats, radioimmunoassay of endogenous TRH extracted from the different regions of the cerebellum shows that it is highly concentrated in the flocculonodular region and paraflocculi, as well as CN, while its concentration in the cerebellar hemispheres and vermis is very low [105]. However, the specific cellular localization of TRH remains unclear. In addition, not both of the two TRH receptors, TRHR1 and TRHR2, has been detected in the cerebellum. In the rat cerebellum, *Trhr2* mRNA is mainly expressed in the GrC layer and interneurons scattered throughout the molecular layer [106,107], whereas *Trhr1* mRNA has not been detected within the cerebellum [108].

The role of TRH in cerebellar development is mainly indirectly through thyroid hormone (TH). TH binds to its intranuclear receptors to regulate the transcription of target genes, which is indispensable for cerebellar development [109]. In primary cerebellar culture, TH augments dendrite arborization and neurite growth of PCs through integrin αvβ3 [110]. Hypothyroidism during the early postnatal period causes aberrant cerebellar morphogenesis. Drug-induced hypothyroid animal models show a prolonged cell proliferation in the EGL and retarded EGL disappearance, a retarded cell differentiation in the molecular layer and IGL, a decreased dendrite arborization of PCs, and a myelination delay and synaptic disconnections among cerebellar neurons and afferent fibers [109]. Moreover, in the human cerebellum, high levels of TRH immunoactivity are detected in the extracts of the fetal cerebellum, and they decline dramatically after 28 weeks of gestation [111], indicating a direct role for TRH in the development of human fetal cerebellum.

Functionally, microiontophoretic application of TRH shows a potent inhibitory effect on the activity of nearly half of the neurons extracellularly recorded in the rat cerebellar cortex [112]. In contrast, subcutaneous injection of TRH increases the firing rate of neurons in the cerebellar interposed nucleus of the mouse cerebellum [108]. In addition, cerebellar TRH also plays a crucial role in synaptic plasticity and motor learning. Electrophysiological evidence shows the absence of LTD at the PF–PC synapses in TRH knockout mice, which can be rescued by bath application of TRH [113]. Moreover, TRH knockout mice show impaired motor learning in the rotarod test, which can be rescued by intraperitoneal injection of TRH [113]. In adult transgenic mice overexpressing dominant-negative TH receptors specifically in cerebellar PCs, LTD-inductive stimulation causes LTP at PF–PC synapses [114]. The mutant mice also display impairments in motor coordination and motor learning [114].

In the clinic, TRH has been used as a treatment for cerebellar ataxias [115]. It has been reported that intraperitoneal injection of TRH improves the ataxic gait of rolling mouse Nagoya (RMN), a mouse model of hereditary ataxia and cerebellar atrophy [116]. Rovatirelin, a newly synthesized TRH analogue, has an ameliorating effect on ataxic symptoms of RMN [117]. The underlying mechanism may be related to an elevated uptake of glucose in the cerebellum and an increase in cerebellar *Bdnf* mRNA levels [117]. In addition, TRH receptor agonists also significantly ameliorate 3-AP-induced cerebellar ataxia in rats, and the effect of TRH is completely counteracted by NMDA receptor antagonists [118]. More importantly, intravenous administration of TRH for two weeks significantly improves clinically evaluated cerebellar ataxia in patients with spinocerebellar degeneration [119,120], particularly in lessening dysmetria [115]. Recently, resveratrol and related polyphenols have been reported to have therapeutic effects on many neuropsychiatric and neurodegenerative diseases, and cerebellar TRH and TRH-like peptides may be potential mediators of the therapeutic actions of these polyphenols [121]. However, the mechanism for anti-ataxic and neuroprotective actions of TRH has not yet been well determined and requires more intensive exploration.

## 8. Oxytocin

Oxytocin, a cyclic nonapeptide neurohormone synthesized primarily by magnocellular neurons within the paraventricular and supraoptic nucleus of the hypothalamus, was successfully sequenced in 1953, and is known for its uterine-contracting and lactation-promoting effects through the hypothalamo-neurohypophyseal system [122,123]. In addition, the hypothalamic oxytocinergic neurons project extensively to extra-hypothalamic target regions to exert a broad spectrum of reproductive and social processes via oxytocin receptor (OXTR) [123]. In the cerebellum, the localization of oxytocin is inconsistent across species. Oxytocin-immunoreactivity fibers have been observed within the cerebellar white matter and to extend to some extent into the GrC layer and cerebellar fastigial nucleus in rats [124], whereas they are located in the molecular layer but not the GrC layer in mice [125] (Figure 1). Despite the discrepancy of oxytocin in anatomical expression among species, the spatial distribution of OXTR in the cerebellum is relatively restricted [126,127], suggesting a common role of oxytocin signaling in the cerebellum. OXTR staining is mainly found in the somata of neurons with very little expression in fibers [124]. OXTR is exclusively expressed in PCs in the mouse cerebellar cortex, especially in the lobule Crus I [128] (Figure 1). Although oxytocin plays a vital role in brain development [129] and social interactions [130], no developmental changes or sex differences in OXTR expression have been found in the cerebellum [127,128].

Several lines of evidence based on human functional magnetic resonance imaging (fMRI) studies have shown that oxytocin delivered nasally may affect functional connectivity between the cerebellum and other brain areas. The cerebellum could influence affective and cognitive-related forebrain structures associated with ASD via cerebello-thalamo-cortical loops and cerebello-thalamo-basal ganglia loops [81]. Oxytocin decreases cerebellar–putamen connectivity in healthy men [131], and enhances cerebellar–posterior cingulate cortex connectivity in participants who experience low levels of maternal love withdrawal [132]. Several lines of evidence suggest that the connections between the cerebellum and forebrain structures are vulnerable to ASD genetic risks [133,134]. Abnormalities in the oxytocin signaling associated with ASD have also been reported [135]. However, whether oxytocin can modulate cerebellar activity and functions directly remains ambiguous. In awake rats, intraperitoneal injection of oxytocin activates the cerebellum, whereas oxytocin given directly to the brain has no significant impact on the blood oxygen level-dependent (BOLD) signal in the cerebellum, indicating that peripheral oxytocin may influence the cerebellar activity indirectly through a peripheral oxytocin signaling pathway. Consistently, in cerebellar slice preparations, bath application of oxytocin does not affect firing activity, intrinsic excitability, or synaptic transmission of PCs of not only normal rats but also the maternal immunoactivation (MIA) mouse model of autism [128], whose occurrence and development are considered closely related with oxytocin and cerebellum. Furthermore, blockage of OXTR in Crus I in wild-type mice does not induce autistic-like social, stereotypic, cognitive, or anxiety-like behaviors [128]. These results suggest that oxytocin signaling in Crus I PCs seems to be independent of ASD pathophysiology. Notably, as a G protein-coupled receptor, OXTR activation may exert a long-term effect on gene transcription of PCs via the regulation of cAMP-responsive element binding protein (CREB) and CREB-regulated transcriptional coactivators (CRTC) or myocyte enhancer factor 2 (MEF-2) transcription factor complex [136,137]. Disturbances of CREB and MEF-2 pathways are both ASD-related risk factors [138,139]. It has been reported that oxytocin administration significantly increases the mRNA expression levels of synaptic adhesion molecules in primary cerebellar cultures [140]. Therefore, further elucidation of the effects of oxytocin-induced changes in cerebellar gene transcription may pave a path to uncover the functional relevance of oxytocin and OXTR in the cerebellum.

## 9. Secretin

Secretin, one of the gut peptides with 27 amino acid residues, was initially described as a gut hormone regulating gastrointestinal functions when first discovered in 1902 [141]. After that, using the techniques of radioimmunoassay, immunohistochemical, and in situ hybridization staining, secretin and its receptors have also been discovered in several regions of the central nervous system, including the cerebellum [142] (Figure 1). Secretin immunoreactivity can be detected in the somata and proximal dendrites of PCs. In contrast, secretin receptors are mainly localized in the somata of PCs and GrCs [143,144], including not only the immature GrCs of EGL [143] but also the mature GrCs of IGL [144]. In addition, secretin receptors are also expressed in basket cells within the molecular layer [145], as well as neurons in the CN [144,146].

Both secretin and its receptors are upregulated during the earliest postnatal stages of mice (on P4, P7, and P10 compared against P28) and downregulated from P14, but persist throughout the postnatal period [143]. Recent studies have demonstrated an essential role of secretin in cerebellar neurogenesis and development. Mice with secretin deprivation exhibit an alternation of developmental patterns of PCs and GrCs, resulting in a lower density and dendritic complexity of PCs and a thinner EGL [143]. On brain slices of the developing cerebellum in mice, secretin treatment can promote critical antiapoptotic elements, such as Bcl-2 and Bcl-xL, indicating that secretin may prevent apoptosis during cerebellar development [147].

The secretinergic system also plays a crucial modulatory role in synaptic transmission in the cerebellum. Patch clamp recordings have shown that secretin facilitates spontaneous, evoked, and miniature inhibitory postsynaptic currents (IPSCs) of PCs [145]. Considering that immunoreactivity for secretin is found in the somata and dendrites of PCs and its receptors are expressed in both PCs and basket cells, it has been proposed that secretin may be released from the somatodendritic regions of PCs and serve as a retrograde messenger modulating GABAergic afferent input activity [145,148]. When PCs are excited by a high concentration of potassium or depolarizing current stimulation, an increased release of secretin and an elevated frequency of spontaneous IPSCs on PC somata can be observed [149]. This modulation of secretin on the GABAergic neurotransmission may be mediated by AMPA receptors localized on the presynaptic terminals of basket interneurons [149]. In addition, several behavioral studies have revealed the role of secretin in cerebellum-dependent learning and memory, indicating a possible involvement of secretin in regulating synaptic plasticity in the cerebellum. Both exogenous and endogenous cerebellar secretin can promote the acquisition phase of delay eyeblink conditioning by reducing surface expression of potassium channel Kv1.2 α subunit in PC dendrites and basket cell terminals of rats [150,151]. Moreover, specific knockout of secretin in PCs significantly impairs mouse motor coordination and motor learning [146]. However, whether secretin has a direct impact on cerebellar synaptic plasticity remains unknown.

Widespread interest has focused on secretin as a treatment for autism. In recent decades, a growing number of studies have focused on cerebellar nonmotor functions, in particular their relationship with ASD [81]. Abnormal development and physiology of the cerebellum during the sensitive period of early life may account for key autistic features [81]. As mentioned above, secretin is critical to the morphogenesis and function of the cerebellum during development [143]. Some observations on clinical cases have shown that secretin administration significantly alleviates the symptoms of children with ASD, manifested by increased eye contact and improved verbal communication and attention [152,153]. However, other studies have failed to find any evidence to support the effectiveness of secretin in the treatment of autism [154]. Moreover, no evidence has been reported for a causal relationship between human secretin gene mutation and autism [155]. Therefore, whether secretin holds a critical position in autism pathology and intervention remains enigmatic and needs to be further studied.

## 10. Conclusions

Although the peptidergic system has not traditionally been incorporated in the cerebellar model and framework, findings over the past decade suggest crucial roles for neuropeptides in the cerebellar neurogenesis and development, neuronal excitability and activity, as well as synaptic transmission and plasticity modulation, thus actively participating in the regulation of cerebellar somatic motor and nonsomatic (nonmotor) functions, including emotion and cognition, and particularly somatic–nonsomatic integration, summarized in Table 2. In addition to cerebellar physiological functions, altered levels of cerebellar neuropeptides have been implicated in the pathophysiology and pathogenesis of cerebellum-related disorders, such as ataxias and autism. Although neuropeptides are rarely used to treat cerebellar ataxias and autism, their involvement in cerebellar pathophysiology shows excellent translational potential in real-world clinical settings. The eight reviewed neuropeptides most associated with neurodegenerative and neuropsychiatric disorders and derivatives are favored as novel, potentially promising agents in forthcoming neuropharmacology. Therefore, advances in functional studies of the cerebellar neuropeptides may provide a novel framework for a better understanding of the physiology and pathology of the cerebellum and pave a new path to develop novel potential therapeutic strategies for treating neurodegenerative and neuropsychiatric disorders.

## Figures and Tables

**Table 1:** Major cerebellar neuropeptides that have attracted the most attention in the last two decades.

Order	Neuropeptide Name	Abbreviation	No. of Publications Available on PubMed in Last Two Decades (2004–2024)	Potential Clinical Treatment
1	Brain-derived neurotrophic factor	BDNF	403	SCA6, SCA1
2	Insulin-like growth factor 1	IGF-1	110	Metabolic diseases
3	Corticotropin-releasing factor (hormone)	CRF (CRH)	68	Ataxias
4	Angiotensin II	Ang II	49	ASD
5	Somatostatin	SS	45	
6	Cerebellin	CBLN	41	
7	Neuropeptide Y	NPY	40	SCA3
8	Orexin	OX	33	Narcolepsy-cataplexy, anxiety
9	Thyrotropin-releasing hormone	TRH	32	Ataxias
10	Oxytocin	OXT	29	ASD
11	Calcitonin-gene-related peptide	CGRP	28	Migraine
12	Substance P	SP	26	
13	Secretin	SCT	24	ASD
14	Cholecystokinin	CCK	22	
15	Dynorphin	DYN	17	

Note: Eight neuropeptides with gray background color are the most closely related to neurodegenerative and/or neuropsychiatric disorders, such as ataxia, ASD, narcolepsy–cataplexy, anxiety, etc. Abbreviations: SCA, spinocerebellar ataxia; ASD, autism spectrum disorder.

**Table 2:** Roles of neuropeptides in cerebellar physiology and pathophysiology.

	Cerebellar Development	Neuronal Excitability, Synaptic Transmission, and Synaptic Plasticity	Molecular, Circuital and Behavioral Phenotype
**BDNF**	Survival, migration, maturation, and arborization of GrCs ↑ [21,22,23,24,25,26] Survival of PCs ↑ [36] CF synapse elimination ↑ [27] Inhibitory synapse development ↑ [25,27]	GrC and PC excitability ↑ [28] GABAergic transmission in PCs ↑↓ [29,30,31] Ionic plasticity ↑ [32] Paired-pulse facilitation ↑ [33]	Neuroprotective effect ↑ [22,37] Neuroimmune modulation ↑ [38,39] Ataxic gait ↓ [23,36]
**CRF**	PC development ↑ [64,65] Presynaptic development in the cerebellar cortex ↑ [62,63]	Glutamatergic neuron excitability in the interposed nucleus ↑ [43] PC excitability ↑ [47] LTD of PF–PC transmission ↑ [48] LTD of CF–PC transmission ↑ [49] LTP of CF–PC transmission ↑ [50] LTD between interneuron and PC ↓ [51] LTP of MF–GrC transmission ↑ [52]	Motor control under challenging conditions ↑ [44] Motor performance ↑ [43] Motor learning ↓ [52,57] Ataxic symptoms ↓ [43]
**Angiotensin II**	IO–cerebellar pathway development ↑ [73] Neurite outgrowth and cell migration ↑ [74]	PC excitability ↓ [75] GABAergic transmission in PCs ↑ [75]	Aging- and senescence- related gene expression ↑ [70] *Il-10* mRNA expression ↓ [76] Cyclooxygenase 2 expression ↑ [77] Proinflammatory effect ↑ [77] Astrocyte mitosis ↑ [77] Systemic oxytocin levels ↑ [80]
**NPY**	Cerebellar volume ↑ [89] GrC survival ↑ [89]		BDNF levels ↑ [89] Anti-inflammatory effect ↑ [89] Mutant ataxin-3 aggregates ↓ [89] Motor coordination ↑ [89,90]
**Orexin**		CN excitability ↑ [98,102] Vestibular nuclear complex excitability ↑ [99] PC excitability ↑ [100]	Cerebellar theta oscillations ↑ [101] The timing of TEC ↑ [101] Motor performance during a motor challenge ↑ [99] Arterial blood flow redistribution ↑ [100] Anxiolytic effect ↑ [102]
**TRH**	Migration and differentiation of GrCs ↑ [109] Dendritogenesis and neuritogenesis of PCs ↑ [109,110] Synaptogenesis ↑ [109]	Neuronal excitability in the cerebellar cortex ↓ [112] Neuronal excitability in the interposed nucleus ↑ [108] LTD of PF–PC transmission ↑ [113,114]	*Bdnf* mRNA levels ↑ [117] Glucose uptake ↑ [117] Motor coordination ↑ [114] Motor learning ↑ [113,114] Ataxic symptoms ↓ [115,116,117,118,119,120]
**Oxytocin**			Synaptic adhesion molecule ↑ [140] Cerebellum-putamen connectivity ↓ [131] Cerebellum-posterior cingulate cortex connectivity ↑ [132]
**Secretin**	Survival and arborization of PCs ↑ [143] Survival and maturation of GrC ↑ [143] Anti-apoptotic effects [147]	GABAergic transmission in PCs ↑ [145,148,149]	The acquisition phase of DEC ↑ [150,151] Motor coordination ↑ [146] Motor learning ↑ [146] Autistic-like behaviors ↓ [152,153]

Note: “↑” indicates positively regulated by the corresponding neuropeptides, “↓” indicates negatively regulated, and “↑↓” indicates regulated bidirectionally through different downstream molecules. Abbreviations: DEC, delay eyeblink conditioning; TEC, trace eyeblink conditioning.

## Data Availability

Not applicable.
